# Existem Maneiras Alternativas para Estimar a Atividade Inflamatória Aterosclerótica de Pacientes com Síndrome Coronariana Aguda?

**DOI:** 10.36660/abc.20220492

**Published:** 2022-08-25

**Authors:** Alexandre de Matos Soeiro

**Affiliations:** 1 Instituto do Coração Universidade de São Paulo Unidade de Emergência São Paulo SP Brasil Instituto do Coração, Universidade de São Paulo - Unidade de Emergência, São Paulo, SP – Brasil; 2 Hospital BP Mirante São Paulo SP Brasil Hospital BP Mirante, São Paulo, SP – Brasil

**Keywords:** Aterosclerose/prevenção e controle, Placa Aterosclerótica,/ tratamento farmacológico, Inflamação/sangue, Inflamação/tratamento farmacológico, Síndrome Coronariana Aguda, Agentes Anti-Inflamatórios/uso terapêutico

O tema da inflamação presente no processo aterosclerótico vem sendo alvo de inúmeras pesquisas há anos. Definitivamente, a aterosclerose é uma doença inflamatória, uma vez que o acúmulo de leucócitos no subendotélio é um dos primeiros processos que ocorrem na formação da placa. Posteriormente, outras células inflamatórias (monócitos, macrófagos, células dendríticas, linfócitos e mastócitos) participam continuadamente desde a formação da “estria gordurosa”, até a ocorrência do evento coronariano agudo.^1 - 3^

A ação orquestrada de todos os sinais pró-inflamatórios que existem na placa não só aumenta a inflamação, como também afeta diretamente os elementos estruturais que suportam sua estabilidade mecânica. Uma variedade de mensageiros pró-inflamatórios são liberados por células endoteliais imunológicas e vasculares, ativando citocinas, quimiocinas, compostos lipídicos bioativos e moléculas de adesão que mantêm e aceleram a inflamação e o desenvolvimento local de lesões ateroscleróticas.^4 , 5^

Dentre todos os marcadores inflamatórios relacionados à aterosclerose, a proteína-C reativa (PCR) tem sido a mais utilizada na prática clínica. Níveis de PCR podem muitas vezes fornecer informações úteis para diagnosticar, tratar e monitorar pacientes com aterosclerose, e confirmar as respostas do paciente a vários fatores estimulantes. Alguns estudos apontam que a PCR se liga ao LDL e está presente nas placas ateroscleróticas. A PCR não está normalmente presente na parede do vaso saudável, mas torna-se detectável nos estágios iniciais da aterogênese e se acumula durante a progressão da aterosclerose. A PCR é considerada um preditor de eventos cardiovasculares futuros, e na população geral os níveis de PCR são capazes de prever o risco de forma independente de mortalidade cardiovascular.^4^

Além da PCR, outros marcadores clássicos foram estudados e apresentam relação direta com o processo aterosclerótico. Dentre eles, destacam-se a interleucina-6, interleucina-1, moléculas de adesão (P-selectina, L-selectina, ICAM-1, VCAM-1 e PECAM-1) e metaloproteinases (MMPs).^4 , 6 , 7^ Atualmente discute-se o papel de células mortas em apoptose, anticorpos contra fosfolípides, proteínas *heat shock* , infecções na placa ( *Chlamydia pneumoniae. Porphyromonas gingivalis, Aggregatibacter actinoomycetemcomitans, Helicobacter pylori* e Citomegalovírus).^6^

Apesar da enorme extensão de dados na literatura, a maioria dos estudos explora apenas a doença aterosclerótica crônica. Em pacientes com síndromes coronarianas agudas, as informações são escassas. No estudo apresentado, os autores avaliaram o índice imunoinflamatório sistêmico (IIS) como um marcador prognóstico em quadros agudos. Dito índice, representado pela relação entre plaquetas x neutrófilos/contagem de linfócitos, foi aplicado em 309 pacientes e correlacionado com a carga aterosclerótica e complicações hospitalares. Os neutrófilos classicamente estão relacionados a processos inflamatórios agudos e seu aumento expressivo possivelmente eleva o IIS em quadros mais graves. De fato, IIS mais elevados foram observados em pacientes com maior tempo de internação, com escore Syntax mais elevado, com maiores valores de troponina e com síndrome coronariana aguda com supradesnível de ST. Portanto, trata-se de um índice de fácil reprodutibilidade e que pode alertar para maior atividade inflamatória no processo aterosclerótico agudo vigente, refletindo gravidade.^8^

Apesar da atividade inflamatória da aterosclerose ser bem descrita, terapias anti-inflamatórias capazes de atingir a inflamação ainda estão faltando no arsenal do médico. A maioria dos ensaios clínicos de ‘anti-inflamatórios’ foram negativos, embora o estudo CANTOS ( *Canakinumab Anti-inflammatory Thrombosis Outcomes Study* ) utilizando canakinumab provou que bloquear a via da interleucina-1β pode reduzir eventos cardiovasculares.^9^ Alvos mais novos são necessários e a genética pode auxiliar na resolução desse problema.^1^
