# Supplementary material for: Existem Maneiras Alternativas para Estimar a Atividade Inflamatória Aterosclerótica de Pacientes com Síndrome Coronariana Aguda?
Source: Arq Bras Cardiol. 2022 Aug 25;119(3):391–2. [Article in Portuguese] doi: 10.36660/abc.20220492 (PMC9438537; doi:10.36660/abc.20220492)
Supplement: Translation

# Translation: Are There Alternative Ways to Estimate Atherosclerotic Inflammatory Activity in Patients with Acute Coronary Syndrome?

**Authors:** Alexandre de Matos Soeiro

**Affiliations:** 1 Brazil Instituto do Coração, Universidade de São Paulo - Unidade de Emergência, São Paulo, SP – Brazil; 2 Brazil Hospital BP Mirante, São Paulo, SP – Brazil

**Keywords:** Atheroclerosis/prevention and control, Plaque, Atherosclerotic/drug therapy, Inflammation/blood, Inflammation/drug therapy, Acute Coronary Syndrome, Anti-Inflammatory Agents/therapeutic use

The theme of inflammation in the atherosclerotic process has been the subject of numerous researches for years. Atherosclerosis is an inflammatory disease since the accumulation of leukocytes in the subendothelium is one of the first processes in plaque formation. Subsequently, other inflammatory cells (monocytes, macrophages, dendritic cells, lymphocytes and mast cells) participate continuously from forming the “fatty groove” until the occurrence of the acute coronary event.^1 - 3^

The orchestrated action of all the pro-inflammatory signals in the plaque increases inflammation and directly affects the structural elements that support its mechanical stability. Various pro-inflammatory messengers are released by immune and vascular endothelial cells, activating cytokines, chemokines, bioactive lipid compounds and adhesion molecules that maintain and accelerate inflammation and the local development of atherosclerotic lesions.^4 , 5^

C-reactive protein (CRP) has been the most used in clinical practice among all inflammatory markers related to atherosclerosis. CRP levels can often provide useful information for diagnosing, treating and monitoring patients with atherosclerosis and confirming the patient’s responses to various stimulating factors. Some studies indicate that CRP binds to LDL and is present in atherosclerotic plaques. CRP is not normally present in the healthy vessel wall but becomes detectable in the early stages of atherogenesis and accumulates during the progression of atherosclerosis. CRP is considered a predictor of future cardiovascular events, and in the general population, CRP levels can independently predict the risk of cardiovascular mortality.^4^

In addition to CRP, other classical markers were studied and are directly related to the atherosclerotic process. Among them interleukin-6, interleukin-1, adhesion molecules (P-selectin, L-selectin, ICAM-1, VCAM-1 and PECAM-1) and metalloproteinases (MMPs) stand out.^4 , 6 , 7^ Currently, the role of dead cells in apoptosis, antibodies against phospholipids, heat shock proteins, plaque infections ( *Chlamydia pneumoniae. Porphyromonas gingivalis, Aggregatibacter actinoomycetemcomitans, Helicobacter pylori and Cytomegalovirus* ) is being discussed.^6^

Despite the enormous amount of data in the literature, most studies only explore chronic atherosclerotic disease. In patients with acute coronary syndromes, information is scarce. In the presented study, the authors evaluated the systemic immunoinflammatory index (SII) as a prognostic marker in acute conditions. This index, represented by the relationship between platelets x neutrophils/lymphocyte count, was applied in 309 patients and correlated with atherosclerotic burden and in-hospital complications. Neutrophils are classically related to acute inflammatory processes, and their significant increase possibly elevates SII in more severe cases. In fact, higher SII was observed in patients with longer hospital stays, with higher Syntax scores, higher troponin values and acute coronary syndrome with ST-elevation. Therefore, it is an easily reproducible index that can alert to greater inflammatory activity in the current acute atherosclerotic process, reflecting severity.^8^

Although the inflammatory activity of atherosclerosis is well described, anti-inflammatory therapies capable of targeting inflammation are still lacking in the physician’s arsenal. Most clinical trials of ‘anti-inflammatory’ were negative, although CANTOS (Canakinumab Anti-inflammatory Thrombosis Outcomes Study) using canakinumab proved that blocking the interleukin-1β pathway can reduce cardiovascular events.^9^ Newer targets are needed, and genetics can help solve this problem.^1^
